# Trophectoderm, Inner Cell Mass, and Expansion Status for Live Birth Prediction After Frozen Blastocyst Transfer: The Winner Is Trophectoderm

**DOI:** 10.3390/life14111360

**Published:** 2024-10-23

**Authors:** Alessandro Bartolacci, Sofia de Girolamo, Lisett Solano Narduche, Elisa Rabellotti, Lucia De Santis, Enrico Papaleo, Luca Pagliardini

**Affiliations:** 1 Obstetrics and Gynaecology Unit, IRCCS San Raffaele Scientific Institute, Via Olgettina, 60, 20132 Milan, Italy; degirolamo.sofia@hsr.it (S.d.G.); rabellotti.elisa@hsr.it (E.R.); desantis.lucia@hsr.it (L.D.S.); papaleo.enrico@hsr.it (E.P.); pagliardini.luca@hsr.it (L.P.); 2 Reproductive Sciences Laboratory, Obstetrics and Gynaecology Unit, IRCCS San Raffaele Scientific Institute, Via Olgettina, 60, 20132 Milan, Italy; solano.lisett@hsr.it

**Keywords:** blastocyst, trophectoderm, inner cell mass, expansion status, live birth

## Abstract

Despite advancements in technologies such as time-lapse microscopy and artificial intelligence, the gold standard for embryo selection still relies on standard morphological assessment. Several studies have investigated the correlation between blastocyst characteristics (expansion status, inner cell mass, and trophectoderm) and clinical outcomes, reaching contradictory results. In consideration of these ambiguities in the literature, we performed a retrospective study of 1546 untested first-vitrified–warmed single day 5/6 blastocyst transfers. The purpose of our study is to evaluate three scenarios: (i) independent association between each morphological characteristic (expansion status, inner cell mass, and trophectoderm) and live birth; (ii) comparison between blastocysts with inner cell mass grade A and trophectoderm grade B and blastocysts with inner cell mass grade B and trophectoderm grade A; and (iii) comparison between poor-quality day 5 and top-quality day 6 blastocysts. After adjusting for principal confounders, we report that trophectoderm is more predictive of live births than inner cell mass and expansion status. We observed a trend in favor of top-quality day 6 blastocysts over poor-quality day 5 blastocysts. Moreover, on the same day of development and expansion status, blastocyst BA should be preferable to blastocyst AB.

## 1. Introduction

In vitro fertilization (IVF) treatments have revolutionized the field of reproductive medicine, offering hope to couples dealing with infertility challenges. The main goal of IVF is to facilitate a single healthy live birth [1]. To enhance the success of this goal, the most effective approach seems to be performing a single blastocyst transfer. Moreover, in recent years, vitrification has become the preferred method for cryopreserving blastocysts in human IVF, resulting in a high live birth rate [2,3]. Blastocyst selection is a crucial step in the IVF process. Despite advancements in certain technologies, such as time-lapse microscopy and artificial intelligence (AI), the gold standard for embryo selection still relies on standard morphological assessment [4,5]. To aid in this selection process, a widely accepted morphological classification system was introduced by Gardner and Schoolcraft over a decade ago [6]. This system classifies blastocysts based on three criteria: the degree of expansion and hatching; the quality of the inner cell mass (ICM); and the trophectoderm cells (TE). Numerous studies and expert consensus support the validity of this classification system [7,8,9]. The morphological quality of blastocyst characteristics is closely associated with clinical outcomes [10,11,12,13,14,15,16]. Nevertheless, the superiority of either of these morphological features is still under debate. Several studies have investigated the correlation between ICM, TE, expansion status (ES), and clinical outcomes, reaching contradictory results [10,11,12,13,14,15,16]. Previous studies showed ICM as the best predictor for implantation and clinical pregnancy compared to TE, suggesting ICM as a biomarker for viability [11,12,17]. In contrast, several studies have reached a different conclusion, asserting that the TE quality is the superior predictor of reproductive competence in euploid and untested blastocysts [14,15,16,18]. In addition, evidence suggests that ES is a predictor of live birth (LB) after single blastocyst transfer [12].

Gaining a comprehensive understanding of predictive power of individual morphological characteristics, such as ICM, TE, and ES, is crucial in embryo transfer selection. While the Istanbul Consensus did not specifically explore this aspect, it did suggest that the ICM may have greater significance in predicting the implantation rate [19]. However, current evidence does not distinguish between the relative clinical performance of ICM grade A and TE grade B blastocysts and ICM grade B and TE grade A blastocysts. Moreover, when only poor-quality day 5 and top-quality day 6 blastocysts are available, it can indeed be challenging to determine which should be prioritized [20,21]. These uncertainties underscore the need for further investigation in this area.

In consideration of these ambiguities in the literature, we performed a retrospective study of 1546 untested first-vitrified–warmed single day 5/6 blastocyst transfers. The purpose of our study is to evaluate three scenarios: (i) the independent association between morphological characteristic (ICM, TE, and ES) and LB; (ii) the comparison between blastocysts with ICM grade A and TE grade B and blastocysts with ICM grade B and TE grade A; and (iii) the comparison between poor-quality day 5 and top-quality day 6 blastocysts.

## 2. Materials and Methods

### 2.1. Study Population

This is a retrospective study including 1546 patients who received first-vitrified–warmed single day 5/6 untested blastocyst transfers between January 2019 and October 2023 after first-freeze-all intracytoplasmic sperm injection (ICSI) cycles. Exclusions included (i) ICSI cycles utilizing surgically obtained or previously frozen sperm; (ii) cycles involving donor gametes; and (iii) cycles with oocytes that were previously cryopreserved and thawed. No restrictions criteria were adopted for maternal age.

During the study period (5 years) no changes were introduced in the clinical and laboratory routine. New staff were introduced only after a period of training to assure harmonization with pre-existing operators. Nevertheless, to minimize the risk of bias due to variations between operators, two senior embryologists, each with over six years of expertise, assessed each blastocyst simultaneously. This study was approved by the local Institutional Review Board.

### 2.2. Ovarian Stimulation Protocol, ICSI, and Embryo Culture

All the vitrified–warmed blastocysts used in this study were obtained in stimulated oocyte retrieval ICSI freeze-all cycles as previously described [22]. A daily protocol involving either a GnRH agonist or antagonist was applied for pituitary suppression. Ovarian stimulation was carried out using highly purified FSH (IBSA, Lodi, Italy), with the initial dose ranging from 100 to 300 IU per day, based on the patient’s hormonal profile and body measurements. Both initial and adjusted doses were personalized based on individual responses to gonadotropins. Final oocyte maturation was triggered with HP–human chorionic gonadotropin (hCG) (Gonasi; Amsa, Rome, Italy) once one or more follicles reached a diameter of 17 mm or greater. Oocyte retrieval was conducted 35–36 h post-hCG trigger through transvaginal ultrasound-guided aspiration.

ICSI was conducted as previously described [22]. Fertilization was assessed 16–18 h after the procedure, with normal fertilization confirmed by the presence of two pronuclei (2PN) and two polar bodies. Embryos were systematically cultured to the blastocyst stage on day 5. In cycles with extended culture, the culture medium (Sage In-Vitro Fertilization, Inc., Trumbull, CT, USA) was refreshed on day 3 by moving embryos into a newly prepared dish on day 2.

### 2.3. Blastocyst Morphological Assessment

Blastocyst morphology was evaluated using a modified version of the Gardner and Schoolcraft criteria [23]. In summary, on the morning of days 5 to 6 of development, blastocysts were graded based on the following: (i) the degree of expansion and hatching, with scores ranging from 1 (early blastocyst) to 5 (hatched blastocyst). (ii) The assessment of ICM, where “A” is characterized by a prominent, well-compacted, and easily identifiable group of cells. These cells are numerous, tightly packed, and cohesive. “B” indicates fewer small cells forming a non-continuous uneven layer with some gaps. “C” represents a poorly defined small group of cells, with few cells forming the aggregate and a lower degree of organization. (iii) The evaluation of the TE, where “A” represents many identical small cells forming a tightly knit continuous epithelium, “B” indicates fewer small cells forming a non-continuous uneven layer with some gaps, and “C” represents a sparse number of cells of uneven size forming a loose epithelium with more gaps. According to modified Gardner and Schoolcraft [23], a top-quality blastocyst was defined as a fully expanded or hatching blastocyst with an “A” grade for the ICM and the TE, or with one component graded “A” and the other “B”. Blastocysts were considered good quality if both the ICM and TE were graded “B”, or if one was “B” and the other “C”. Poor-quality blastocysts were those with ICM and TE rated as “C”.

In our clinic, all embryologists responsible for embryo assessment were rigorously trained, qualified, and experienced. Morphological grading is subjective and qualitative; therefore, intra- and interlaboratory variations may exist. Nevertheless, to minimize the risk of bias due to variations between operators, two senior embryologists, each with over six years of expertise, assessed each blastocyst simultaneously. In cases of disagreement, the decision was taken in agreement with the IVF laboratory director. The operators assessing the blastocysts included in this study were compared for their consistency in the grading of ICM, TE, and ES on a set of 50 randomly chosen blastocysts. The resulting interclass correlation coefficients (ICC) were 0.85, 0.80, and 0.82, respectively.

### 2.4. Blastocyst Vitrification and Warming

Expanded blastocysts were cryopreserved using the simplified embryo vitrification protocol developed by Irvine Scientific (https://www.irvinesci.com/) after artificial shrinkage. The equilibration step was conducted at room temperature for 10 min. Subsequently, the blastocysts were moved into three 50 µL drops of vitrification solution for 1 min before loading. A single blastocyst was frozen using an individual storage device. The thawing process followed the simplified embryo warming protocol from Irvine Scientific (https://www.irvinesci.com/) using the Irvine Vit Kit-Thaw (FUJIFILM Irvine scientific, Santa Ana, CA, USA). Once thawed, the blastocysts were transferred to a culture dish containing six 30 µL drops of blastocyst medium (Sage In-Vitro Fertilization, Inc., Trumbull, CT, USA) beneath 3 mL of mineral oil (Sage In-Vitro Fertilization, Inc., Trumbull, CT, USA). The culture dishes were prepared the day before the FBTs and incubated overnight in a controlled environment with 6% CO_2_ and 5% O_2_.

### 2.5. Embryo Transfer Procedure

Single vitrified–warmed untested blastocyst transfers were performed on day 5 or day 6 according to the guidelines of the American Society for Reproductive Medicine [24]. All frozen blastocyst transfers (FBTs) were performed via either a natural or an artificial cycle. The blastocysts remained in the culture drops until the time of transfer (2 to 3 h); subsequently, they were transferred into a one-well culture dish with 1.5 mL of blastocyst medium (Sage In-Vitro Fertilization, Inc., Trumbull, CT, USA). Two operators, the transfer operator and the witness operator, moved the blastocyst into the one-well culture dish before loading the embryo into the catheter. All FBTs were performed with ultrasound guidance, as previously described [25].

### 2.6. Outcome Measure

The primary outcome of the study was the LB. The LB rate was defined as the number of live births out of the total number of transferred blastocysts.

### 2.7. Statistical Analysis

Data analysis was performed with the Statistical Package for Social Sciences (SPSS) version 26.0 (SPSS Inc., Chicago, IL, USA). All continuous variables were expressed as means ± standard deviation (SD). For prediction of the LB, multivariate analysis was conducted using generalized linear models (GLM); this approach allows for the adjustment of treatment correlations within individuals. The number of retrieved oocytes, inseminated oocytes, fertilized oocytes, mature oocytes, total motile sperm count (million), maternal age, paternal age, days of stimulation, estrogen on the day of the ovulation trigger, progesterone concentration on the day of the ovulation trigger, cause of infertility, and transfer preparation protocols were included as confounding variables in the GLM model to obtain adjusted estimation for the effect of ES, ICM, and TE grading on LB. The predictive power of blastocyst morphology was investigated by comparing LB in three scenarios: (i) independent association between each morphological characteristic (expansion status, inner cell mass, and trophectoderm) and live birth; (ii) comparison between blastocysts with inner cell mass grade A and trophectoderm grade B and blastocysts with inner cell mass grade B and trophectoderm grade A; and (iii) comparison between poor-quality day 5 and top-quality day 6 blastocysts.

To test the hypothesis that TE are stronger predictors of LB than ICM and ES, a receiver operating characteristic (ROC) curve analysis was also performed. All the reported *p*-values were corrected for confounders, as previously described, and a *p*-value < 0.05 was considered statistically significant.

## 3. Results

A total of 1546 first vitrified–warmed day 5/6 blastocyst transfer cycles were included in this study. Cycle baseline characteristics of FBTs are reported in Table 1. Overall, the LB rate is 26.1%, while on day 5, blastocyst transfer is 32.7%, and on day 6, blastocyst transfer is 19.5%.

After adjusting for principal confounders, TE were independently associated with LB in overall and day 6 blastocyst transfers (Table 2).

When the TE grade dropped from A to C, the likelihood of achieving a LB was reduced from 37.9% to 18.3% [OR = 1.370, 95%IC (1.117–1.680), adjusted *p* = 0.002, overall blastocyst transfer] and from 40.6% to 14.3% [OR = 1.711, 95% CI (1.238–2.366), adjusted *p* = 0.001, day 6 blastocyst transfer] (Table 2). 

**Figure 1:**
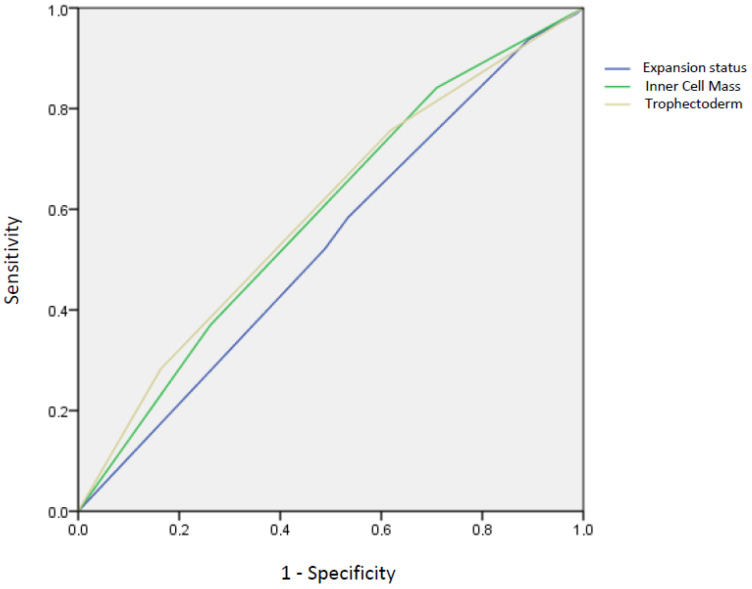
Live birth prediction according to ROC curves derived from expansion status [AUC = 0.529 (95% CI 0.497–0.561), *p*-value = 0.08], inner cell mass [AUC = 0.587 (95% CI 0.555–0.619), *p*-value < 0.001], and trophectoderm [AUC = 0.595 (95% CI 0.563–0.627), *p*-value < 0.001].

In line with multivariate logistic regression, the ROC curve analysis indicated that TE are more accurate than ICM and ES in predicting LB [TE: AUC = 0.595 (95% CI 0.563–0.627), ICM: AUC = 0.587 (95% CI 0.555–0.619), ES: AUC = 0.529 (95% CI 0.497–0.561), overall blastocyst transfer] (Figure 1). 

Blastocyst ICM grade A and TE grade B presented a significantly lower LB chance than blastocyst ICM grade B and TE grade A (AB); the probability of achieving an LB was increased from 32.1% (AB) to 40.9% (BA) [OR = 1.751, 95% CI (1.010–3.038), adjusted *p* = 0.046, overall blastocyst transfers] (Table 3). A trend, though not statistically different, was found in favor of day 6 top-quality blastocysts over day 5 poor-quality blastocysts [33.9% Vs 28.2%, OR= 1.147, 95% CI (0.453–2.898), *p*-value = 0.773], especially considering only AA as top-quality on day 6 [41.4% Vs 28.2%, OR = 1.270, 95% CI (0.507–3.184), *p*-value = 0.608] (Table 4).

## 4. Discussion

Current evidence has not conclusively defined the best predictor of LB among blastocyst features (ES, ICM, and TE). To provide new insights in this field, we performed a retrospective study including 1546 patients receiving the first-cryopreserved single day 5/6 blastocyst transfers (n = 772 for day 5 blastocysts; n = 774 for day 6 blastocyst). Our study aims to determine the best predictor of LB among ICM, TE, and ES. Several studies have investigated the correlation between blastocyst quality and clinical outcomes, reaching contradictory results [10,11,12,14,15,16,17,18]. After adjusting for principal confounders, we report that TE were independently associated with LB. In contrast to our results, previous studies have suggested ICM as a biomarker of viability [11,17]. Generally, ICM was the most significant characteristic affecting transfer outcomes, maybe because it fated to become fetal tissue, while TE developed into the placenta. On the other hand, according to our results, several studies showed that TE cells are a stronger predictor of clinical outcomes [10,14,16,18,26]. TE cells, responsible for forming the placenta, are essential for implanting into the endometrium and maintaining pregnancy. One study demonstrated that TE cells produce prostaglandins, which have a well-established role in facilitating embryo–endometrium interaction [27]. Additionally, a previous study found that prostaglandin H synthase is abundantly expressed in the TE, while only minimal background activity was detected in the ICM [28]. This highlights the potential importance of the TE, rather than the ICM, in mediating interactions with the endometrium at the implantation site. A previous study showed that TE quality is significantly related to ongoing pregnancy and miscarriage rates [18]. Taken together, these results may speculate that the implantation of TE into the endometrium is a critical step in sustaining pregnancy and obtaining a LB. In line with multivariate logistic regression, our ROC curve analysis demonstrated that TE are more accurate than ICM and ES in predicting LB. 

Interestingly, Alfarawati et al. showed that the decline in TE quality (from A to C) corresponded to an increased incidence of aneuploidy [29]. Specifically, they found that the likelihood of aneuploidy is 2.5 times higher in embryos with a TE grade of C compared to those with a TE grade of A. 

Moreover, we investigated the predictive potential of blastocysts with ICM grade A and TE grade B compared to those with ICM grade B and TE grade A, as well as the outcomes between poor-quality day 5 blastocysts and top-quality day 6 blastocysts. Our goal was to provide key insights to assist embryologists in selecting the optimal blastocyst for warming. Interestingly, the probability of achieving an LB was increased from 32.1% (AB) to 40.9% (BA) [OR = 1.751, 95% CI (1.010–3.038), adjusted *p* = 0.046]. Although blastocysts with ICM grade A and TE grade B (AB) and those with ICM grade B and TE grade A (BA) are both classified as top-quality blastocysts according to the Istanbul consensus [19], our data revealed a significant difference in live birth (LB) rates between these two qualities (AB vs. BA). This finding is crucial in selecting the most viable blastocyst for transfer. Moreover, these results reinforce the findings of previous studies which suggest that trophectoderm is the most reliable biomarker for predicting blastocyst viability [14,15,18].

A recent study demonstrated that top-quality day 6 blastocysts are preferred over poor-quality day 5 blastocysts when selecting blastocyst transfer [21]. Moreover, one study with a large sample size found no significant difference in pregnancy and perinatal outcomes between the delayed formation of high-quality expanded blastocysts on day 6 and those formed on day 5, regardless of their quality [20]. Our findings align with those of Shi and colleagues, showing a trend favoring top-quality day 6 blastocysts, albeit not statistically significant. However, the limited sample size in our study restricts the strength of this analysis. Therefore, further investigation is needed to fully evaluate the differences between poor-quality day 5 blastocysts and top-quality day 6 blastocysts. We are currently planning an extension of this study to address this gap. 

One limitation of standard morphological assessment is its subjectivity, which can lead to intra- and interlaboratory variations. An interesting study showed that the morphological assessment of embryos can vary among different embryologists within the same center and also between different centers [30]. It is important to recognize the growing relevance of advanced non-invasive techniques such as time-lapse imaging and artificial intelligence (AI). These methods offer promising opportunities to enhance embryo selection by providing continuous monitoring and objective assessments that could complement traditional grading criteria. These technologies can reduce interoperator variability and improve reproducibility [31].

However, in our study, to minimize this potential bias, all operators assessing the blastocysts were compared for their consistency in grading the ICM, TE, and ES across a set of 50 randomly chosen blastocysts. This comparison resulted in high intraclass correlation coefficients (ICCs) for all blastocyst features (ES, ICM, and TE), indicating a high level of consistency.

Additionally, a recent manuscript introduced an innovative method for standardized TE assessment. This new approach makes objective blastocyst assessment feasible, allowing for standardization across operators and laboratories worldwide [32].

Our study assumes relevance in the context of clinical practice.

When selecting a blastocyst for warming, a day 5 blastocyst should be prioritized when the morphology is equal. Blastocyst selection should follow the classifications AA, BA, and AB. Additionally, if only poor-quality day 5 and top-quality day 6 blastocysts are available, our data have revealed a trend, although not statistically significant, favoring the use of top-quality day 6 blastocysts (AA, AB, BA) (33.9% vs. 28.2%, OR= 1.147, 95% CI (0.453–2.898), *p*-value = 0.773). This trend increases considering only day 6 AA (41.4% vs. 28.2%, OR = 1.270, 95% CI (0.507–3.184), *p*-value = 0.608). However, these data should be interpreted with caution due to the small sample size analyzed (n = 69 blastocysts).

Moreover, while our study supports TE quality as a stronger predictor of implantation and clinical success, it is important to discuss the clinical implications of selecting embryos with high-quality TE but poor-quality ICM, especially given that the ICM contributes directly to fetal tissue development.

Our findings, prioritizing TE quality over ICM quality, might understandably raise concerns about the potential risks of transferring blastocysts with lower ICM quality. However, recent studies have demonstrated that poor blastocyst quality does not necessarily increase the risk of adverse perinatal outcomes [33]. This suggests that selecting embryos based primarily on TE quality is clinically sound, even in cases where ICM quality is suboptimal. Therefore, prioritizing embryos with superior TE quality, despite lower ICM quality, is unlikely to compromise perinatal outcomes. In contrast, selecting an embryo with a high ICM but poor TE quality could reduce the chances of successful implantation and pregnancy establishment.

Taken together, these data support the idea that prioritizing TE quality is a reasonable approach, without significant risk associated with transferring embryos that may have lower ICM quality.

One limitation of this study is that we did not assess cumulative live births. Another limitation is the retrospective nature of this study. However, despite its retrospective nature, this study is strengthened by its analysis of a large number of first FBTs from the first ICSI freeze-all cycles. Furthermore, by focusing exclusively on FBTs, we eliminated any potential differences due to endometrial asynchrony, which could occur in fresh cycles.

## 5. Conclusions

In conclusion, our findings showed that TE cells are the strongest predictor of live birth in day 5/6 untested vitrified warmed blastocyst transfer. We observed a trend in favor of top-quality day 6 blastocysts over poor-quality day 5 blastocysts. Moreover, when comparing blastocysts at the same stage of development and ES, blastocyst BA should be prioritized over blastocyst AB. However, further randomized controlled trials (RCTs) are necessary to validate our results.

## Figures and Tables

**Table 1:** Patient demographics and cycle parameters for untested blastocyst transfer by live birth outcome.

	Untested Blastocyst Transfer (n = 1546)		*p*-Value
	Live Birth	No Live Birth	
Number of cycles	403	1143	n.a.
Maternal age at oocyte retrieval, years (mean ± SD)	34.38 ± 3.29	36.15 ± 3.79	<0.001
Maternal age at transfer, years (mean ± SD)	34.86 ± 3.35	36.69 ± 3.86	<0.001
Paternal age at oocyte retrieval, years (mean ± SD)	37.92 ± 5.28	39.04 ± 5.55	0.001
AMH (ng/mL), mean ± SD	3.72 ± 2.62	3.13 ± 2.50	<0.001
Progesterone (ng/mL), mean ± SD	1.02 ± 0.77	1.03 ± 0.55	0.543
Day of stimulation, mean ± SD	10.12 ± 3.62	10.02 ± 2.23	0.895
Number of retrieved oocytes, mean ± SD	12.76 ± 6.56	11.37 ± 6.25	<0.001
Mature oocytes, mean ± SD	9.51 ± 4.05	8.77 ± 3.96	<0.001
Cause of infertility, n (%)			
Male factor	96 (23.82)	290 (25.37)	0.536
Endometriosis	31 (7.69)	64 (5.60)	0.133
Tubal factor	20 (4.96)	55 (4.81)	0.904
Unexplained infertility	6 (1.49)	17 (1.49)	0.998
Poor ovarian reserve	24 (5.96)	53 (4.64)	0.296
mixed	226 (56.08)	664 (58.09)	0.482
Expansion grade, n (%)			0.055
1	5 (1.24)	19 (1.66)	
2	20 (4.96)	105 (9.18)	
3	143 (35.48)	410 (35.87)	
4	26 (6.45)	51 (4.46)	
5	209 (51.86)	559 (48.90)	
ICM grade, n (%)			<0.001
A	149 (36.97)	299 (26.15)	
B	190 (47.14)	512 (44.79)	
C	64 (15.88)	332 (29.04)	
TE grade, n (%)			<0.001
A	114 (28.28)	187 (16.36)	
B	191 (47.39)	520 (45.49)	
C	98 (24.31)	436 (38.14)	

n.a., not applicable; n, number; SD, standard deviation; ng/mL, nanograms/milliliters; AMH, anti-Müllerian hormone; ICM, inner cell mass; TE, trophectoderm.

**Table 2:** Blastocyst characteristics (expansion status, inner cell mass, and trophectoderm according to modified Gardner’s criteria) and live birth.

	**Expansion Status (n = 1546 Overall; n = 772 Day 5; n = 774 Day** **6)**						
	**1**	**2**	**3**	**4**	**5**	**Adjusted OR (95% CI)**	***p*-Value**
Live birth, overall blastocyst, n (%)	5/23 (21.7)	20/125 (16.0)	143/553 (25.9)	26/77 (33.8)	210/768 (27.3)	1.091 (0.979–1.216)	0.115
Live birth, day 5 blastocyst, n (%)	1/7 (14.3)	15/59 (23.7)	97/297 (32.7)	12/34 (35.3)	128/375 (34.1)	1.110 (0.962–1.282)	0.153
Live birth, day 6 blastocyst, n (%)	4/16 (25.0)	5/66 (7.6)	46/256 (18.0)	14/43 (32.69	82/393 (20.9)	1.081 (0.910–1.284)	0.376
	**Inner Cell Mass (n = 1546 Overall; n = 772 Day 5; n = 774 Day** **6)**						
	**A**		**B**	**C**		**Adjusted OR (95% CI)**	***p*-Value**
Live birth, overall blastocyst, n (%)	150/448 (33.5)		190/702 (27.1)	64/396 (16.2)		1.146 (0.938–1.400)	0.184
Live birth, day 5 blastocyst, n (%)	123/354 (34.7)		113/351 (32.2)	16/69 (23.2)		1.098 (0.828–1.456)	0.517
Live birth, day 6 blastocyst, n (%)	27/94 (28.7)		77/351 (21.9)	48/327 (15.0)		1.033 (0.754–1.416)	0.840
	**Trophectoderm (n = 1546 Overall; n = 772 Day 5; n = 774 Day** **6)**						
	**A**		**B**	**C**		**Adjusted OR (95% CI)**	***p*-Value**
Live birth, overall blastocyst, n (%)	114/301 (37.9)		192/711 (27.0)	98/534 (18.3)		1.370 (1.117–1.680)	0.002
Live birth, day 5 blastocyst, n (%)	86/232 (37.1)		128/423 (30.3)	38/117 (32.5)		1.106 (0.840–1.458)	0.472
Live birth, day 6 blastocyst, n (%)	28/69 (40.6)		64/288 (22.2)	60/418 (14.3)		1.711 (1.238–2.366)	0.001

OR, Odd ratio; CI, confidence interval; All analyses adjusted for confounders.

**Table 3:** Live birth of blastocyst ICM grade A, TE grade B (AB), and ICM grade B, TE grade A (BA).

	Adjusted OR (95% CI)	*p*-Value
Live birth overall blastocyst, BA (n = 88) vs. AB (n = 227)	1.751 (1.010–3.038)	0.046
Live birth day 5 blastocyst, BA (n = 53) vs. AB (n = 170)	1.741 (0.874–3.468)	0.115
Live birth day 6 blastocyst, BA (n = 35) vs. AB (n = 56)	1.659 (0.570–4.831)	0.353

OR, odds ratio; CI, confidence interval. All analyses adjusted for confounders and expansion status. BA: reference group.

**Table 4:** Live birth of poor-quality day 5 and top-quality day 6 blastocysts.

	LB Rate	Adjusted OR (95% CI)	*p*-Value
Live birth top-quality (AA, AB, BA) day 6 (n = 121) blastocysts vs. poor-quality (CC) day 5 (n = 39), %	33.9% vs. 28.2%	1.147 (0.453–2.898)	0.773
Live birth top-quality (AA) day 6 (n = 30) blastocysts vs. poor-quality (CC) day 5 (n = 39), %	41.4% vs. 28.2%	1.270 (0.507–3.184)	0.608

LB, Live birth; OR, odds ratio; CI, confidence interval. All analyses are adjusted for confounders and expansion status.

## Data Availability

The data that support the findings of this study are available from the corresponding author upon reasonable request.
